# First- and Second-Trimester Cardiovascular Anomalies in Trisomy 21 Fetuses: Anatomy, Embryology, Genetics and Imaging

**DOI:** 10.3390/jpm16070358

**Published:** 2026-06-30

**Authors:** Mariangela Pati, Immacolata Blasi, Giovanna Botticelli, Andrea Musarò, Flavio Vanacore, Giulia Galeati, Lorenzo Aguzzoli, Maria Paola Bonasoni

**Affiliations:** 1 Unit of Obstetrics and Gynecologic Oncology, Azienda USL-IRCCS di Reggio Emilia, 42122 Reggio Emilia, Italy; mariangela.pati@ausl.re.it (M.P.); immacolata.blasi@ausl.re.it (I.B.); giovanna.botticelli@ausl.re.it (G.B.); andrea.musaro@ausl.re.it (A.M.); flavio.vanacore@ausl.re.it (F.V.); giulia.galeati3@gmail.com (G.G.); lorenzo.aguzzoli@ausl.re.it (L.A.); 2 Pathology Unit, Azienda USL-IRCCS di Reggio Emilia, 42122 Reggio Emilia, Italy

**Keywords:** trisomy 21, congenital heart disease, fetal echocardiography, gene dosage

## Abstract

**Background**: Trisomy 21 (T21) is strongly associated with congenital heart disease, particularly atrioventricular septal defect (AVSD), ventricular septal defect (VSD), atrial septal defect (ASD) and selected conotruncal and arch anomalies. First- and second-trimester ultrasound, Doppler and fetal cardiac MRI enable increasingly early and detailed characterization of these lesions, while advances in molecular cardiogenesis have linked specific phenotypes to dosage-sensitive genes on chromosome 21. **Methods**: This narrative review synthesizes contemporary evidence on structural and functional cardiovascular anomalies in T21 fetuses in the first and second trimester, integrating fetal echocardiography, Doppler assessment and fetal cardiac MRI with embryologic and molecular insights, and summarizing trimester-specific detectability and pathophysiologic links to candidate genes in the Down syndrome-critical region. Approximately one quarter to one third of T21 fetuses have major congenital heart disease on high-quality prenatal echocardiography, with AVSD representing about half of all lesions and VSD, tetralogy of Fallot (TOF), arch anomalies, venous return abnormalities and functional markers (increased nuchal translucency, tricuspid regurgitation, ductus venosus abnormalities) comprising the remainder. **Results**: First-trimester detection relies on functional markers and early four-chamber and outflow-tract views, whereas second-trimester studies refine anatomic definition and hemodynamics, with MRI reserved for complex cases. Overexpression of genes such as DSCAM, COL6A1/COL6A2, DYRK1A and RCAN1 perturbs endocardial cushion, conotruncal and vascular development. **Conclusions:** Early, protocol-driven cardiac imaging in T21 supports timely diagnosis, risk stratification and multidisciplinary counselling, and links fetal imaging phenotypes with chromosome 21 gene dosage to advance personalized management and future genotype–phenotype research.

## 1. Introduction

Down syndrome (DS), or trisomy 21 (T21), is the most frequent autosomal aneuploidy, occurring in about 1 in 800 live births, and congenital heart disease (CHD) is present in roughly 35% to 50% of affected children. T21 is linked to a wide range of physical, neurodevelopmental, psychological, and functional comorbidities, which intersect with CHD to shape overall health, functional capacity, and quality of life throughout the lifespan. Over recent decades, advances in cardiac interventions and perioperative care have markedly improved survival for infants and children with CHD, including those with T21 [1]. The American Heart Association (AHA) recommends routine echocardiographic imaging for all fetuses and infants diagnosed with T21 due to the high prevalence and spectrum of CHD [1]. In pregnancy, in the first and second trimester, increased nuchal translucency (NT), abnormal ductus venosus (DV) flow, tricuspid regurgitation (TR) and early signs of hydrops are frequent ultrasound (US) markers that correlate with both T21 and underlying cardiac anomalies. Fetal echocardiography, Doppler ultrasonography and, in selected cases, fetal cardiac magnetic resonance imaging (MRI) complement each other for detailed characterization, especially when acoustic windows are suboptimal or extracardiac anomalies coexist [2,3].

The most common types of CHD in fetuses with T21 are atrioventricular septal defect (AVSD), ventricular septal defect (VSD), and atrial septal defect (ASD). Tetralogy of Fallot (TOF) and patent ductus arteriosus (PDA) occur less commonly, while aortic arch abnormalities, coarctation of the aorta, and anomalous venous return are rare in this population [1,4]. Specifically, AVSD accounts for about 24% of cases detected prenatally in large cohorts, and in aggregate, AVSD, VSD, and ASD constitute roughly 70% of congenital heart disease cases in T21 [1]. TOF is less common than AVSD, VSD, and ASD, but it remains a recognized lesion, estimated between 2% and 6% [1]. In some populations, VSD may be the most common lesion, with reported rates up to 39%, followed by patent ductus arteriosus (34%), ASD (23%), and AVSD (16%) [5]. These variations reflect differences in population demographics and diagnostic practices.

This review summarizes the most common CHD in DS, with a particular focus on anatomy, embryology, and genetic effects of trisomy 21 on the cardiovascular system. The main features of early diagnosis in pregnancy are also described.

This narrative review was developed through a structured, non-systematic search of PubMed/MEDLINE, Scopus, and Web of Science, focused primarily on English-language articles published from 2000 to 2026, with selective inclusion of earlier landmark studies when they provided foundational anatomic, embryologic, or imaging insights not reproduced in later literature. Priority was given to studies addressing first- and second-trimester cardiovascular anomalies in trisomy 21, fetal echocardiography, Doppler assessment, fetal cardiac MRI, genotype–phenotype correlations, and prenatal or postnatal outcome data relevant to counselling.

## 2. Atrioventricular Septal Defect (AVSD)

### 2.1. Anatomy

AVSD is strikingly over-represented in T21, occurring at an approximately 1000- to 2000-fold higher prevalence than in the general CHD population [1].

**Figure 1:**
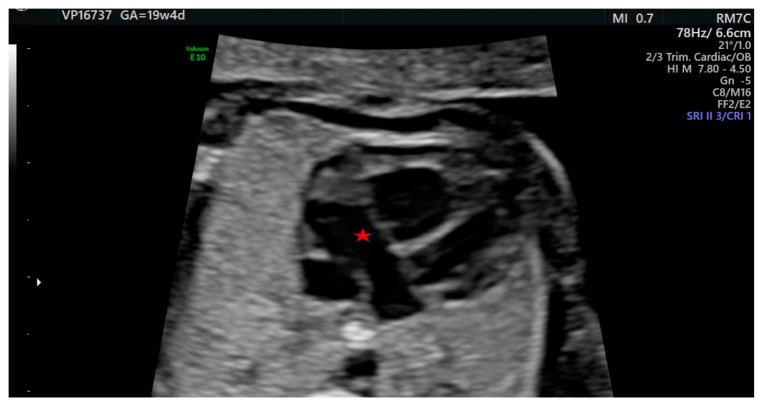
Partial AVSD in a 19-week-Down syndrome fetus: A primum ASD (red star) is seen contiguous with the atrioventricular valve plane.

AVSDs are caused by abnormal development of the endocardial cushions, resulting in deficiencies of the atrial and ventricular septa and a common atrioventricular (AV) junction with characteristic AV valve abnormalities. Classification includes three types: partial, intermediate/transitional, and complete [6]. In the partial form, there is a primum ASD contiguous with the AV valve plane, separate right and left AV valves, and a cleft in the left AV valve, but no true inlet VSD (Figure 1). In the intermediate/transitional type, a primum ASD coexists with a restrictive inlet VSD, while two distinct AV valve orifices are preserved. In the complete variation, there is a common AV junction with a single common AV valve and combined atrial and inlet VSDs, typically producing a large primum ASD contiguous with an inlet VSD and exposure of the crest of the interventricular septum to the common valve [6].

From a morphological standpoint, AVSD is described as balanced or unbalanced depending on the relative commitment of the common AV valve to the right and left ventricles, which determines whether a biventricular or functionally single-ventricle circulation is feasible.

For complete AVSD, the Rastelli classification subdivides the common AV valve into three types (A, B, and C) based on the chordal attachments and degree of “bridging” of the superior (anterosuperior) bridging leaflet across the ventricular septum [6]. The classification is as follows:

- Rastelli type A: the superior bridging leaflet is divided and attached by chordae directly to the crest of the interventricular septum, so that most chordal support remains near the septal crest and the common valve is relatively symmetrically committed to both ventricles; this is the most frequent subtype and is often associated with left-sided obstruction from accessory tissue.
- Rastelli type B: part of the superior bridging leaflet is supported by an anomalous papillary muscle or chordae arising from the right ventricular septal surface, rather than inserting on the septal crest, so that the leaflet “bridges” further into the right ventricle; this configuration is uncommon and is characterized by dominant insertion of anterior leaflets into right-sided papillary muscle.
- Rastelli type C: the superior bridging leaflet has no chordal attachment to the interventricular septum and appears to “float” freely over a large inlet VSD, with extreme bridging into the right ventricle; this subtype is frequently associated with more complex malformations such as TOF and other conotruncal anomalies.

### 2.2. Embryology and Genetics

AVSD reflects abnormal development of the atrioventricular endocardial cushions, mesenchymal cap, and dorsal mesenchymal protrusion, leading to failed septation of the atrioventricular junction [7]. In trisomy 21, overexpression of genes including DYRK1A, DSCAM, and COL6A1/COL6A2 appears to perturb epithelial–mesenchymal transition, extracellular matrix organization, and cushion development, thereby increasing susceptibility to AVSD [8,9,10,11]. Therefore, these dosage-sensitive mechanisms help explain why the predominant prenatal phenotype in trisomy 21 is a defect centered on the atrioventricular junction and endocardial cushions, often visualized as complete or balanced AVSD rather than isolated valve dysplasia.

### 2.3. Imaging

**Figure 2:**
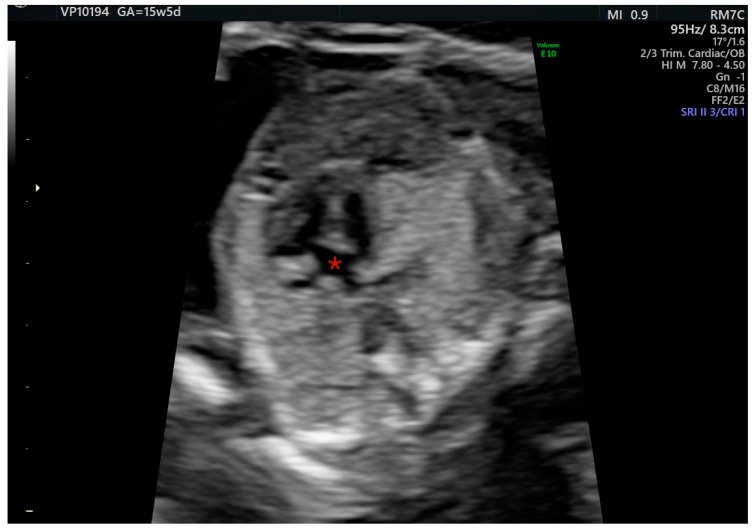
Complete AVSD in a Down syndrome fetus of 15 weeks + 5 days: the atrio-valvular junction is common with combined ASD and VSD (star).

**Figure 3:**
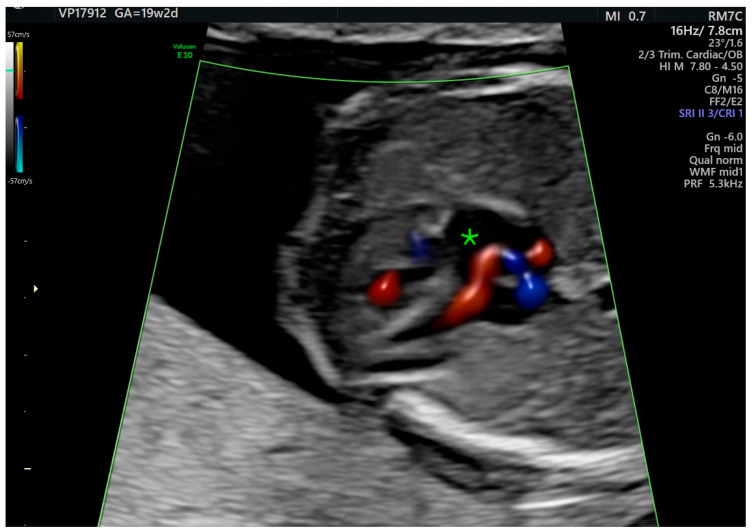
Complete AVSD in a Down syndrome fetus of 19 weeks + 2 days: color Doppler US shows aortic vascular efflux (star) which partially overlap the atrial blood flow.

On first-trimester US, subtle four-chamber abnormalities such as loss of the offset between AV valves, common AV valve appearance, and increased NT may be present; color Doppler can show a single inflow jet through a common valve. In the second trimester, a classic four-chamber view demonstrates a defect in the atrioventricular septum with a common AV valve and frequently balanced ventricular sizes, while Doppler shows AV valve regurgitation and altered inflow patterns [2,12] (Figure 1, Figure 2 and Figure 3). Fetal cardiac MRI can help in assessing ventricular size balance, AV valve morphology and associated lesions (e.g., left ventricular outflow tract obstruction), especially when US windows are poor [3].

## 3. Ventricular Septal Defect (VSD)

### 3.1. Anatomy

VSDs in T21 are most commonly classified by their anatomical location within the interventricular septum: perimembranous, muscular, inlet (atrioventricular canal-type), and outlet (conal septal) defects [13]. VSDs in T21 are most often perimembranous, but all anatomical subtypes can occur, and their prevalence and distribution may vary by population [14]. In Asian cohorts, VSD is the most common CHD associated with T21, whereas AVSD is more prevalent in Caucasian populations [5].

### 3.2. Embryology and Genetics

The embryology of ventricular septal defect (VSD) centers on abnormal development and fusion of the components forming the interventricular septum.

The septum has two main parts: the muscular septum, which develops from the coalescence of trabecular sheets in the primitive ventricle, and the membranous septum, which forms where the ventricular septum meets the atrioventricular junction and outflow tract. Muscular VSDs arise from incomplete coalescence or excessive undermining of these trabecular sheets, whereas perimembranous and inlet VSDs reflect localized failure of closure around the embryonic interventricular foramen at the junction between the muscular septum and the atrioventricular junction. Outlet VSDs occur when formation or fusion of the muscular infundibular sleeve is incomplete, creating continuity between the aortic and pulmonary valves [5,15,16].

Regarding the underlying genetic mechanisms, candidate contributors include dosage-sensitive loci in 21q22.2 and genes such as DSCAM, BACE2, PLAC4, and HMGN1, with additional modifier effects reported for interferon-receptor cluster variants and copy-number changes involving genes such as RIPK4 [9,10,17,18,19,20]. These genetic factors appear less lesion-specific than in AVSD, but they plausibly contribute to septal maldevelopment that presents prenatally as perimembranous, inlet, or mixed septal defects. 

### 3.3. Imaging

First-trimester detection of large perimembranous or inlet VSDs is challenging but feasible with high-resolution transvaginal US, especially when color Doppler is used to highlight abnormal left-to-right shunting across the septum. The American Society of Echocardiography recommends color Doppler interrogation of the septae in all fetal echocardiographic examinations, as it facilitates identification of shunt direction and velocity, and improves detection of septal defects, particularly when 2D image resolution is suboptimal or at earlier gestational ages [2,21]. In the second trimester, perimembranous, inlet, and muscular VSDs can be demonstrated in multiple imaging planes, with color Doppler aiding in the detection of small defects and assessment of shunt direction and velocity [21,22]. The American Society of Echocardiography specifically notes that short-axis views with color Doppler are useful for evaluating the membranous and muscular ventricular septum, and for identifying VSDs and their hemodynamic significance [21]. Clinical studies confirm that color Doppler improves detection rates of isolated VSDs and provides additional information on shunt flow that may not be apparent on B-mode imaging alone [22].

MRI usually has a limited role in isolated small VSD but may contribute in complex lesions or when evaluating global ventricular function and associated extracardiac anomalies [3].

## 4. Atrial Septal Defect (ASD)

### 4.1. Anatomy

ASDs are classified anatomically into four main types: ostium secundum, ostium primum, sinus venosus, and coronary sinus (unroofed coronary sinus) defects [13,23].

Ostium secundum ASD is the most common type, located in the region of the fossa ovalis at the central portion of the atrial septum. It results from deficiency or absence of septum primum tissue and can vary in size and shape, sometimes presenting with multiple fenestrations. The margins are defined by the septum secundum (superior/posterior), AV canal septum (anterior), and septum primum/inferior vena cava valve (inferior) [8,12].

Ostium primum ASD is located at the inferior portion of the atrial septum, adjacent to the atrioventricular valves. It is considered part of the spectrum of AVSDs and is almost always associated with a cleft in the anterior mitral leaflet [8,12].

Sinus venosus ASD is found near the entry of the superior or inferior vena cava into the right atrium. The superior type is more common and is frequently associated with partial anomalous pulmonary venous return [8,12].

Coronary sinus (unroofed coronary sinus) ASD is rare (<1%) and involves a defect in the wall between the coronary sinus and the left atrium, resulting in shunting through the ostium of the coronary sinus [8,12].

### 4.2. Embryology and Genetics

Ostium secundum ASD arises when normal atrial septation fails around the fossa ovalis. Normally, the septum primum grows toward the endocardial cushions, its initial gap (foramen primum) closing as the leading-edge fuses and apoptosis creates the foramen secundum. The septum secundum then forms to the right, partially covering the foramen secundum and leaving the foramen ovale as a flap–valve communication. In ostium secundum ASD, excessive resorption, deficiency, or malformation of the septum primum—or, less often, underdevelopment of the septum secundum—leads to a persistent defect within the true atrial septum at the fossa ovalis, sometimes with multiple fenestrations [23].

Ostium primum ASD arises when the embryonic ostium primum fails to close at the base of the atrial septum, leaving a defect immediately adjacent to the atrioventricular valves and representing the atrial component of the AVSD spectrum described above [23].

Sinus venosus ASD arises from abnormal embryologic development at the junction of the systemic venous inflow and the pulmonary veins, rather than from a deficiency in the true atrial septum. During normal development, the common pulmonary vein is incorporated into the left atrium, and a muscular wall forms to separate the right pulmonary veins from the superior vena cava (SVC) and right atrium. In sinus venosus ASD, there is a deficiency or absence of this wall, resulting in “unroofing” of the right pulmonary veins and anomalous drainage into the SVC or right atrium, with a resulting interatrial communication that is typically posterosuperior to the fossa ovalis [23]. This defect is best considered a venovenous malformation, as the communication occurs outside the boundaries of the true atrial septum, and is often associated with partial anomalous pulmonary venous return (PAPVR) of the right upper or middle pulmonary veins. The American Society of Echocardiography emphasizes that sinus venosus defects are not true atrial septal defects, but rather result from partial or complete absence of the sinus venosus septum between the SVC and right upper pulmonary vein (SVC type) or between the right lower/middle pulmonary veins and the right atrium (IVC type) [24].

The embryology of coronary sinus ASD (unroofed coronary sinus) involves a failure of development of the left atrioventricular fold, resulting in partial or complete absence of the wall (roof) separating the coronary sinus from the left atrium. This creates a direct communication between the left atrium and the coronary sinus, which then drains into the right atrium, producing a left-to-right shunt [25]. During normal cardiac development, the coronary sinus is formed by the incorporation of the left horn of the sinus venosus and is separated from the left atrium by the left atrioventricular fold. Imperfect or absent formation of this fold leads to fenestrations or complete unroofing, manifesting as the defect. This anomaly is often associated with a persistent left superior vena cava (PLSVC), termed Raghib syndrome, due to the embryologic persistence of the left cardinal vein [23]. This defect represents a rare form of atrial communication, with the wall of the coronary sinus within the left atrium being deficient or absent, allowing left atrial blood to enter the coronary sinus and subsequently the right atrium. This embryologic error is distinct from other ASDs, as the defect lies outside the true atrial septum and is best considered an interatrial communication rather than a classic septal defect [24].

In trisomy 21, HMGN1 and DSCAM are the principal dosage-sensitive candidates discussed for septal maldevelopment, although evidence is less specific than for AVSD [9,17,18].

### 4.3. Imaging

Atrial septal defects are diagnosed in the first trimester primarily by high-resolution US, including transvaginal or transabdominal fetal echocardiography, with detailed assessment of the four-chamber view and color Doppler imaging to visualize abnormal flow across the atrial septum. The optimal window for imaging is between 11 and 14 weeks’ gestation, when cardiac anatomy can be sufficiently resolved to detect major septal defects, especially in experienced hands [26,27,28]. Color Doppler is essential for identifying abnormal shunting between the atria, which is the hallmark of an ASD [29].

## 5. Tetralogy of Fallot (TOF) and Conotruncal Defects

### 5.1. Anatomy

The anatomical structure of TOF is defined by four cardinal features [30]:

(1) VSD—typically large and nonrestrictive, most often perimembranous, allowing communication between the right and left ventricles; (2) right ventricular outflow tract obstruction—most commonly due to subpulmonary (infundibular) stenosis, but may also involve valvular or supravalvular pulmonary stenosis; the degree of obstruction is variable and determines clinical severity; (3) overriding aorta—the aortic root is displaced so that it receives blood from both ventricles, sitting directly above the VSD; the extent of override can vary, with the aorta committed to both the right and left ventricles; (4) right ventricular hypertrophy—secondary to increased pressure load from outflow tract obstruction.

Additional anatomical variations may include hypoplasia or stenosis of the pulmonary arteries, anomalous coronary arteries, and associated defects such as atrial septal defect or right aortic arch [30].

The common anatomical variations of TOF in T21 include a high prevalence of associated AVSD, particularly the complete form with a common atrioventricular valve orifice. This combined lesion is much more frequent in T21 than in the general population, with studies reporting that up to 25% of T21 patients with tetralogy of Fallot also have AVSD [5].

### 5.2. Embryology and Genetics

The embryological development of TOF is characterized by abnormal alignment and septation of the outflow tract (OFT) during cardiac morphogenesis. The primary defect is anterocephalad (anterior and cephalad) deviation of the infundibular (outlet) septum, which results in malalignment of the ventricular septum. This malalignment causes a large ventricular septal defect, overriding of the aorta, and obstruction of the right ventricular outflow tract, leading to right ventricular hypertrophy [31].

The underlying embryological processes involve deployment and differentiation of second heart field progenitor cells, OFT elongation and rotation, and OFT cushion and valve morphogenesis. Disruption in these steps—particularly in OFT remodeling and septation—can be caused by defects in myocardial, endocardial, or neural crest cell lineages, as well as abnormal signaling pathways that regulate arterial pole development. Neural crest cells indirectly influence OFT alignment through their role in modulating second heart field development [31].

The genetic factors involved in TOF when it is associated with T21 primarily relate to the presence of an extra copy of chromosome 21, which leads to overexpression of dosage-sensitive genes that disrupt cardiac development. Key genes implicated include HMGN1, which modulates epigenetic regulation and myocardial cell fate, contributing to abnormal valvuloseptal development and septation defects [18]. Other candidate genes on chromosome 21 associated with CHD risk include DSCAM, BACE2, PLAC4, ETS2, ERG, and JAM2, as well as non-coding RNAs such as DSCAM-AS1 and MIR3197, which are thought to influence cardiac morphogenesis and the development of conotruncal defects [17,32,33,34].

Genome-wide association studies and sequencing have also identified additional variants outside chromosome 21, such as mutations in GATA3, KCNH2, ENG, FLNA, and GUSB, which may act as modifiers and increase susceptibility to CHD in DS [35].

Therefore, the prenatal imaging phenotype of overriding aorta, large malalignment VSD, and right ventricular outflow tract obstruction can be viewed as the structural expression of disturbed outflow tract septation superimposed on trisomy 21-related gene dosage effects.

### 5.3. Imaging

**Figure 4:**
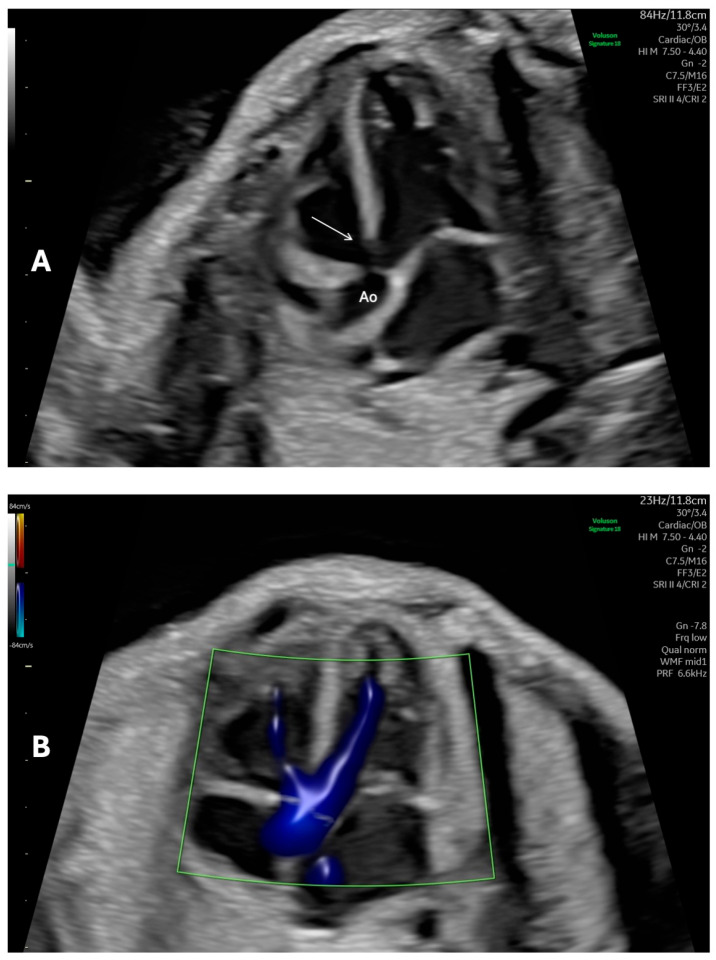
TOF in a Down syndrome fetus of 24 weeks: the aorta (Ao) overrides a complete VSD (arrow) (**A**). (**B**) US color Doppler better shows the overriding aorta (blue vessel) and blood flow from both ventricles.

The approach to diagnosing TOF in the first trimester of pregnancy relies on targeted fetal cardiac imaging during routine US screening at 11–14 weeks’ gestation. The primary modalities are high-resolution transabdominal US, with transvaginal imaging used when fetal position or maternal habitus limits visualization, and adjunctive color Doppler techniques to enhance assessment of flow and vessel alignment [28,36,37,38] (Figure 4).

Key steps include:

1. Systematic evaluation of the four-chamber view, outflow tracts, and three-vessel and trachea (3VT) view. Abnormalities such as a large ventricular septal defect, overriding aorta, and disproportionate vessel sizes (enlarged aortic arch isthmus relative to pulmonary artery) are suggestive of TOF [39,40,41].
2. Use of color Doppler and advanced modalities (e.g., SlowflowHD) to delineate septo-aortic continuity and detect abnormal flow patterns, which improves early detection of conotruncal anomalies [38].
3. Identification of indirect markers, such as increased nuchal translucency or abnormal ductus venosus flow, which prompt referral for early fetal echocardiography in high-risk pregnancies [36,42].

The AHA recommends repeat midtrimester fetal echocardiography for confirmation and detailed anatomical assessment, as image resolution and diagnostic completeness are limited in the first trimester [36].

Fetal cardiac MRI is also valuable for detailed anatomical assessment and as an adjunct to echocardiography in the prenatal diagnosis of TOF, particularly in cases with suboptimal US imaging or complex anatomy [36].

## 6. Coarctation of the Aorta and Aortic Arch Abnormalities

### 6.1. Anatomy

Coarctation of the aorta (CoA) is defined as a congenital narrowing of the aorta, most commonly occurring just distal to the origin of the left subclavian artery, at the site of the ductus arteriosus (juxtaductal region) or adjacent to the ligamentum arteriosum. The American College of Cardiology and AHA describe this as a discrete, short-segment constriction, but variants include long-segment narrowing, arch hypoplasia, or involvement of the aortic isthmus and arch [43].

Hypoplastic aortic arch is characterized by a narrowed and underdeveloped transverse segment of the aortic arch, often with a reduced diameter relative to the ascending aorta. This hypoplasia typically involves the transverse arch between the origin of the left common carotid and left subclavian arteries and may be associated with CoA or other CHDs. The arch may appear as a discrete narrowing or as a diffusely small segment, sometimes resulting in abnormal flow patterns and increased reliance on ductal patency for systemic perfusion [44].

In DS, the most common reported aortic arch anomalies are aberrant aortic arch branching patterns (such as aberrant subclavian artery) and, rarely, vascular rings including double aortic arch [45]. The prevalence of aberrant subclavian arteries, specifically aberrant right subclavian artery (ARSA), in patients with DS is approximately 20–36%, which is markedly higher than the prevalence in the general population (about 1%) [46]. ARSA typically courses posterior to the esophagus and trachea, creating a retroesophageal path that may form a vascular ring and cause symptoms like dysphagia lusoria or respiratory compromise [43,47]. Variants include aberrant left subclavian artery (ALSA) in the context of a right-sided aortic arch, and complex branching patterns such as a bi-carotid trunk or common carotid trunk, which may coexist with aberrant subclavian arteries [48]. All aberrant subclavian arteries have a retroesophageal course, and their presence is associated with increased risk during thoracic surgery and endovascular procedures due to altered anatomy [49].

### 6.2. Embryology and Genetics

CoA arises from a combination of abnormal fetal blood flow distribution, hypoplasia of the aortic arch segments, and tissue remodeling at the juxtaductal region, with the ductus arteriosus playing a critical role in both prenatal compensation and postnatal manifestation of the defect [50].

During normal development, the aortic arch and great vessels arise from the paired pharyngeal (aortic) arches, with contributions from neural crest–derived and vasculogenic cells that remodel into a left-sided aortic arch and descending aorta. Errors in cell migration, differentiation, programmed cell death, and altered biomechanical loading during this remodeling phase have been linked to aortic arch anomalies including isolated CoA. Classic morphogenetic theories for CoA include: (1) extension of ductal tissue from the ductus arteriosus into the juxtaductal aorta, causing constriction as the ductus closes; (2) reduced antegrade left ventricular flow in utero, impairing aortic arch growth and predisposing to left-sided obstructive lesions; and (3) abnormalities of cardiac neural crest cell migration affecting the aortic arch, outflow tract and valves [51,52].

CoA may result from an abnormal developmental process of the left ventral aorta and the fourth–sixth aortic arches, sometimes with persistent or anomalous segments producing discrete narrowing adjacent to the ductus arteriosus [52].

In trisomy 21, NOTCH signaling is intrinsically disturbed in trisomic cells from very early development, leading to abnormal cell programming and angiogenesis that are important for aortic arch formation. Inducible chromosome-silencing and transcriptomic studies in human iPSCs show early dysregulation of NOTCH pathway genes during neural induction [53,54]. Genome-wide analyses implicate NOTCH4 as a risk gene, supporting a role for NOTCH pathway disruption in the excess of structural heart defects in Down syndrome [36].

The embryological factors associated with hypoplastic aortic arch in DS involve disruption of normal aortic arch development, which is dependent on the migration and differentiation of neural crest cells and secondary heart field progenitors. In DS, there is a reduction in ISL1(+) secondary heart field progenitor cells and altered mesodermal development, leading to impaired formation and remodeling of the aortic arch and its branches [34,44]. Abnormal extracellular matrix composition and increased endocardial cushion stiffness further perturb mechanotransduction and cell proliferation during cardiac morphogenesis, contributing to structural anomalies such as hypoplastic aortic arch [55].

Regarding aortic arch abnormalities, ARSA results from abnormal regression of the fourth aortic arch segment, leading to the subclavian artery arising from the descending aorta rather than the brachiocephalic trunk. The aberrant vessel may originate from a dilation known as Kommerell’s diverticulum, which is a remnant of the primitive dorsal aortic arch [43,47,56].

ALSA results from atypical regression and persistence of the left fourth aortic arch and left dorsal aorta [47].

In T21, aortic arch anomalies probably arise from the “generic” arch-patterning machinery (neural crest–second heart field–Notch/TBX1 axis) acting on a heart that is already developmentally stressed by T-21–driven cushion/outflow defects, with T21 genes mainly modulating susceptibility rather than encoding arch-specific patterning factors [10,57].

In practical terms, all these pathways provide a biologic framework for arch hypoplasia, branching anomalies, and altered left-sided growth patterns detected on fetal echocardiography in a subset of T21 fetuses.

### 6.3. Imaging

The approach to diagnosing CoA during the first trimester of pregnancy relies on targeted fetal echocardiography, although sensitivity is limited at this early gestational age. The most informative sonographic parameters include measurement of the aortic isthmus and transverse arch diameters, assessment of the isthmus-to-ductal (I/D) ratio, and evaluation of ventricular and valve size discrepancies. Early signs may include hypoplasia of the distal aortic arch and isthmus, a reduced mitral valve diameter z-score, and increased right-to-left ventricular and pulmonary artery-to-ascending aorta diameter ratios [58,59]. Multiparametric diagnostic models, integrating these anatomical measurements, improve detection rates. For fetuses ≤28 weeks, combining the I/D ratio with the tricuspid/mitral valve ratio (TV/MV) increases diagnostic accuracy, while serial measurements are recommended due to the potential for progressive arch hypoplasia [59]. The use of Z-scores for arch and isthmus diameters, indexed to gestational age, is critical for objective risk assessment [60].

The diagnosis of CoA during the second trimester of pregnancy is primarily based on detailed fetal echocardiography, focusing on anatomical measurements and indices that predict postnatal coarctation. The most sensitive and specific approach is the measurement of the carotid to subclavian artery index (CSAi), with a cut-off value of 0.81 yielding sensitivity of 95.7% and specificity of 99% for coarctation detection [61]. Additional key echocardiographic parameters include the aortic isthmus diameter Z-score, the ductus arteriosus-to-aortic isthmus diameter ratio, and the distal aortic arch (DA) index. A DA index ≥ 1.28 is highly predictive of fetal coarctation cases requiring surgical intervention, with sensitivity of 85% and specificity of 94% [62]. Multiparametric diagnostic protocols, such as a 3-step algorithm incorporating the transverse aortic arch and the descending thoracic aorta (TAO-DAO) angle, transverse arch diameter, isthmus Z-score, and branch distances, achieve diagnostic accuracy above 97% [60]. Other important predictors are the I/D ratio (I/D < 0.74, sensitivity 96.3%; I/D < 0.6, specificity 92.5%), and the TV/MV ratio in fetuses ≤28 weeks, which improve classification of high or low probability of postnatal coarctation [59].

Aortic arch abnormalities are diagnosed during the first and second trimester of pregnancy primarily by fetal echocardiography, using targeted sonographic views. The most critical views are the three-vessel and trachea (3VT) view and the three-vessel view (3VV), which allow visualization of the spatial relationship between the aortic arch, ductus arteriosus, and trachea. Abnormalities are suggested by deviations from the normal “V”-shaped confluence of the aortic and ductal arches, such as a “U”-shaped configuration, size discrepancies, abnormal vessel positions, or separation of the superior vena cava and trachea by intervening arches. These findings can indicate coarctation, right aortic arch, double aortic arch, vascular rings, or ductal aneurysm [21,63]. Color Doppler imaging enhances detection by clarifying flow direction and vessel anatomy. Multi-angle scanning of the aortic arch branches, including the subclavian artery view, improves differential diagnosis, especially for distinguishing right aortic arch from double aortic arch [64]. Aortic arch anomalies can be visualized by the use of advanced techniques such as three-dimensional (3D) and four-dimensional (4D) echocardiography with spatiotemporal image correlation (STIC) and B-flow imaging for detailed assessment of aortic arch anatomy and branching patterns, particularly between 17 and 28 weeks gestation. Specifically, 4D echocardiography with B-flow imaging and STIC was shown to improve visualization of the aortic arch and neck vessels compared to traditional two-dimensional US, with enhanced spatial relationships and anatomical detail. This approach allowed for more accurate prenatal diagnosis of interrupted aortic arch type A and better delineation of the great arteries and their branching patterns during the second trimester (17–28 weeks) as well as later gestation. The use of volume reconstruction in 4D BF-STIC facilitated clinical application and improved diagnostic confidence for aortic arch abnormalities [65].

Key sonographic findings for hypoplastic aortic arch are a small or absent aortic arch, abnormal spatial relationship between the aortic and ductal arches, and discrepancies in vessel diameters. Advanced techniques such as three-dimensional (3D) and four-dimensional (4D) echocardiography with STIC can improve spatial resolution and diagnostic accuracy, especially between 17 and 28 weeks gestation [66].

When fetal echocardiography is inconclusive or limited by fetal position or acoustic windows, fetal MRI provides high-resolution anatomical detail and is unaffected by fetal position, enabling accurate visualization of the aortic arch and its branching patterns [67,68].

## 7. Abnormal Venous Return

### 7.1. Anatomy

The persistent left superior vena cava (PLSVC) is a thoracic venous structure that results from the persistence of the left anterior cardinal vein during embryologic development. It typically begins at the confluence of the left internal jugular and left subclavian veins, descends along the left side of the mediastinum, and courses lateral to the aortic arch. The PLSVC most commonly drains into the right atrium via the coronary sinus, leading to coronary sinus dilation [69].

In most cases, the PLSVC coexists with a right superior vena cava (RSVC), and a bridging innominate vein may connect the two. Rarely, the PLSVC may be isolated (absent RSVC), or may drain directly into the left atrium, which can result in a right-to-left shunt and potential clinical significance [69].

All three anatomical variants—PLSVC with right SVC, isolated PLSVC, and PLSVC draining to the left atrium—are described in patients with T21 [70,71,72].

The incidence of PLSVC in DS is approximately 2–3%—significantly higher than the 0.2–0.5% seen in the general population. This increased incidence is well documented in large echocardiographic series and is associated with CHD, especially AVSDs [73].

Total anomalous pulmonary venous connection (TAPVC) is a congenital defect in which all pulmonary veins drain into the systemic venous circulation rather than the left atrium, requiring an atrial communication for survival. The anatomical structure involves a common pulmonary venous confluence that connects abnormally to systemic veins, bypassing the left atrium [74,75].

TAPVC is classified into four anatomical types based on the site of drainage [75]: (1) Supracardiac: Pulmonary veins drain via a vertical vein into the innominate vein, superior vena cava, or azygos vein (most common, ~50–55%); (2) Cardiac: Pulmonary veins drain directly into the right atrium or via the coronary sinus (~15–30%); (3) Infracardiac: Pulmonary veins drain below the diaphragm, typically into the portal vein, hepatic veins, or inferior vena cava (~13–26%); (4) Mixed: Pulmonary veins drain into multiple sites, combining features of the above types (rare, <10%).

Physiologically, TAPVC is further classified as obstructed or non-obstructed, depending on whether there is significant restriction to pulmonary venous flow at any point in the anomalous pathway [74,75].

Partial anomalous pulmonary venous connection (PAPVC) is defined by one or more, but not all, pulmonary veins draining into a systemic vein or the right atrium instead of the left atrium. The most common anatomical structure involves the right upper pulmonary vein connecting to the superior vena cava, often associated with a sinus venosus atrial septal defect. Other variants include right pulmonary veins draining to the inferior vena cava (as seen in scimitar syndrome), left upper pulmonary vein draining to the left innominate (brachiocephalic) vein, or less commonly, anomalous connections to the coronary sinus or directly to the right atrium [76,77,78].

### 7.2. Embryology and Genetics

The embryological origin of a PLSVC is the failure of the left anterior cardinal vein to regress during embryonic development. Normally, the left anterior cardinal vein involutes and forms the ligament of Marshall, while the right anterior cardinal vein persists as the normal right superior vena cava. When the left anterior cardinal vein does not obliterate, it remains as a persistent left superior vena cava, which typically drains into the right atrium via the coronary sinus [69].

Overexpression of ETS2 and ERG genes, located on chromosome 21, disrupts secondary heart field development, which is critical for proper venous formation [17,34].

The embryological origin of TAPVC and PAPVC is the failure of the common pulmonary vein to develop or connect properly to the left atrium. During normal embryogenesis, the pulmonary venous plexus initially drains into the systemic venous system (cardinal and splanchnic veins). As development progresses, the mid-pharyngeal endothelial strand (MPES) forms and lumenizes, creating the common pulmonary vein, which then connects the pulmonary venous plexus to the left atrium. If the common pulmonary vein fails to form, is atretic, or regresses, the pulmonary veins retain their embryonic connections to systemic veins, resulting in TAPVC, where all pulmonary veins drain into systemic venous structures rather than the left atrium [79].

The genetic factors associated with TAPVC and PAPVC in DS include CNVs and SNPs in candidate regions on chromosome 21 (such as DSCAM, BACE2, PLAC4, ZBTB21, RIPK4), and variants in genes involved in cardiac morphogenesis and left-right patterning (such as GATA3, NOTCH, VEGFA). The risk is polygenic and heterogeneous, with environmental factors also playing a role [17,32,35,79,80]. Although current evidence remains limited, these mechanisms support the concept that abnormal venous return in trisomy 21 is not merely incidental but may reflect broader disturbance of venous pole and pulmonary venous development.

### 7.3. Imaging

Imaging findings for PLSVC during the first and second trimester of pregnancy include the identification of an additional vascular structure to the left of the pulmonary artery in the three-vessel view (3VV) or three-vessel-and-trachea view (3VT) on fetal ultrasound. The four-chamber view (4CV) often reveals a dilated coronary sinus, which is a key indirect sign. High-definition flow render mode and spatiotemporal image correlation (STIC) enhance visualization, allowing dynamic assessment and classification of the vessel’s course and drainage pattern [81,82,83]. When ultrasound findings are equivocal or maternal factors limit image quality, fetal MRI with steady-state free-precession and single-shot turbo spin echo sequences provides adjunctive confirmation and anatomical detail, especially in cases with associated anomalies or poor sonographic windows [84].

Imaging findings for anomalous pulmonary venous connection during the first and second trimester of pregnancy include the absence of pulmonary veins entering the left atrium, a smooth posterior wall of the left atrium, and a small left atrium on the four-chamber view. Additional clues are ventricular disproportion (right ventricle larger than left), increased retroatrial distance between the left atrium and descending aorta, and visualization of a venous confluence or vertical vein posterior to the left atrium on axial views. Color and spectral Doppler imaging can demonstrate abnormal flow patterns, such as continuous turbulent flow in a vertical vein or monophasic continuous flow in pulmonary veins, especially in obstructed cases [4,85,86].

Diagnostic approaches rely on targeted fetal echocardiography, using two-dimensional, color, and spectral Doppler modalities to trace pulmonary venous connections. The three-vessel and trachea view, as well as sagittal and coronal planes, are essential for identifying abnormal venous drainage. Advanced techniques such as three- and four-dimensional ultrasonography, high-definition flow imaging, and spatiotemporal image correlation (STIC) improve visualization and diagnostic accuracy, particularly for complex or partial anomalous connections [86,87,88].

## 8. Summary of Imaging Findings

To clarify trimester-specific detectability, the key lesions reviewed are summarized below according to the imaging findings that are most informative in the first versus second trimester (Table 1).

## 9. Summary of Genetic Pathways

**Figure 5:**
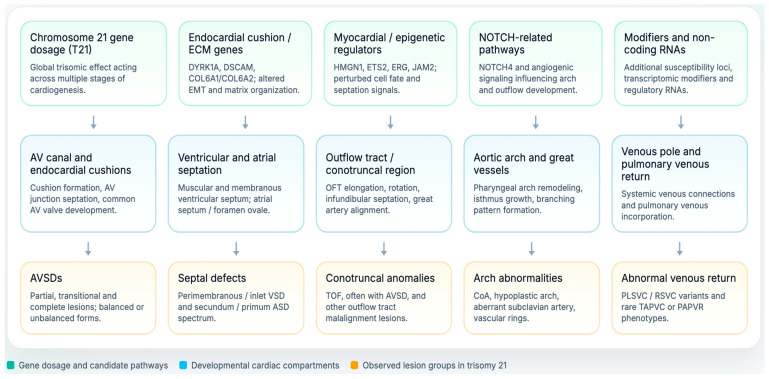
Summary of genetic findings in CHD in Down syndrome.

Figure 5 shows the embryologic pathways that link T21 to CHD.

## 10. Discussion

Prenatal diagnosis of CHD in fetuses with T21 has moved from late to systematic early detection through combined first-trimester markers, NIPT, and targeted fetal echocardiography, enabling accurate delineation of anomaly type and severity and supporting guideline-driven perinatal planning and parental decision-making [26,36]. Prenatal counselling should provide individualized information on prognosis, surgical options, neurodevelopment, and long-term quality of life, in line with AHA recommendations that emphasize natural history, intervention potential, and expected outcomes for each specific cardiac and genetic diagnosis [36,89]. Surveys of fetal cardiologists show that repair is routinely presented as an appropriate postnatal option and that counselling increasingly centers on anticipated functional status and survival rather than karyotype alone [90,91]. Contemporary cohort, database, and multicenter studies indicate that timely surgery in Down syndrome does not substantially increase perioperative mortality, most patients attain good long-term functional status despite higher respiratory and infectious complications, and multidisciplinary counselling—integrating all relevant specialties and psychological support—reduces decisional conflict, lowers termination rates for operable CHD, increases pregnancy continuation, and improves postnatal prognosis [89,92,93,94].

Precise prenatal characterization of congenital heart lesions—including ventricular balance, valve morphology, and associated extracardiac anomalies—allows stratified perinatal planning, guiding delivery timing and mode, prostaglandin E1 use, early transfer, and prioritization of early repair for major shunt lesions to prevent pulmonary vascular disease [21,94,95,96,97]. Lesion-specific counselling is particularly important. Complete and balanced AVSD generally supports planned delivery in a tertiary center and early biventricular repair in infancy, whereas unbalanced AVSD or AVSD combined with TOF may imply more complex surgical decision-making and higher morbidity. Isolated small VSDs or secundum ASDs usually have a more favorable immediate postnatal course and may not require neonatal intervention, while significant coarctation, obstructed venous return, or complex conotruncal disease may require urgent neonatal assessment, prostaglandin support, or early surgery [1,5,58,59,60,61,62,69,70,71,72,73,74,75].

Detailed fetal echocardiographic evaluation and risk stratification are central to coordinated perinatal care and improved outcomes In fetuses with Down syndrome, a normal detailed second-trimester scan has a high negative predictive value for critical CHD, yet targeted fetal echocardiography still identifies relevant non-critical lesions in some cases, which supports AHA recommendations for routine postnatal echocardiographic screening in all neonates with T21 irrespective of prenatal findings [1,95]. Large series, especially from resource-limited settings, show that delays in diagnosis and surgical access markedly increase morbidity and mortality, highlighting the need to strengthen neonatal screening pathways, standardize evaluation protocols, secure equitable access to congenital cardiac surgery, and address social determinants of health and regional disparities in prenatal detection that continue to limit optimal outcomes [96,97].

Genome-wide studies now support a genetically heterogeneous architecture for AVSD and other septal defects in Down syndrome, in which common trisomic dosage genes such as DYRK1A, DSCAM, COL6A1/2 and HMGN1 interact with rare variants and modifier loci, providing a basis for more personalized recurrence counselling, particularly in families with more than one affected child [10,11,18,80,98].

By and large, this lesion-based approach also improves the clinical value of prenatal imaging. AVSD and TOF usually allow relatively direct counselling regarding likely cardiac repair pathways, whereas coarctation and some venous anomalies require counselling that explicitly includes residual diagnostic uncertainty and the possibility of postnatal reclassification [43,58,59,60,61,62,63,64,65,66]. Integrating imaging phenotype with genotype-informed mechanisms may therefore support a more personalized counselling framework, even though current data remain insufficient for deterministic prediction at the individual level [10,17,18,34,36,53,54].

Recent work suggests that artificial intelligence (AI) and machine learning (ML) may increasingly support decision-making in congenital heart disease by integrating imaging, clinical, and biologic data into more precise diagnostic and prognostic models. As reviewed by Pozza et al. [99], AI applications in congenital heart disease already extend beyond image interpretation alone and include risk prediction, computational modelling, telemonitoring, phenotype stratification, and support for personalized follow-up pathways. In the specific setting of trisomy 21, these tools could be especially relevant because prenatal counselling often requires the simultaneous interpretation of multiple variables, including first-trimester screening markers, detailed fetal echocardiographic findings, extracardiac anomalies, and the heterogeneous postnatal course of lesions such as AVSD, VSD, TOF, and aortic arch abnormalities. AI-assisted models could help synthesize these multidimensional data and improve lesion classification, estimate the probability of associated anomalies, refine risk stratification, and support more individualized counselling regarding delivery planning, early neonatal management, and expected surgical pathways. Pozza et al. [99] also emphasize that the future value of AI in congenital heart disease will likely depend on multimodal integration, combining advanced imaging with genetic information, longitudinal clinical data, and remote monitoring, which aligns closely with the personalized medicine perspective relevant to fetuses with trisomy 21. At the same time, the authors note important limitations, including small and heterogeneous datasets, limited external validation, challenges in interpretability, and ethical concerns related to bias, privacy, and overreliance on automated outputs. For this reason, AI should presently be viewed as an adjunct to expert fetal cardiology assessment rather than a replacement for it, but it represents a promising framework for future prenatal models that link genotype, imaging phenotype, and outcome in trisomy 21-associated congenital heart disease.

## 11. Conclusions

This review highlights that T21 is associated with a characteristic spectrum of CHD in which septal, conotruncal, aortic arch, and venous anomalies converge on disrupted endocardial cushion biology, second heart field and neural crest development, and dosage-sensitive overexpression of chromosome 21 genes within a genetically heterogeneous background. By integrating detailed anatomical, embryological, and molecular insights with current advances in first- and second-trimester imaging, including targeted fetal echocardiography and selected fetal cardiac MRI, it underscores how early, precise lesion characterization can guide risk stratification and evidence-based perinatal management.

## Figures and Tables

**Table 1:** Summary of the imaging findings.

Lesion/Pattern	First Trimester: Most Informative Markers	Second Trimester: Most Informative Markers	Typical Role of MRI
**AVSD**	Increased NT, abnormal DV flow, TR, loss of AV valve offset, common inflow jet on color Doppler.	Defect at the atrioventricular septum, common AV valve, AV valve regurgitation, ventricular balance assessment.	Adjunctive in complex AVSD or poor acoustic windows; useful for ventricular balance and associated lesions.
**VSD**	Large perimembranous or inlet defects may be suspected with high-resolution imaging and color Doppler shunting.	Multiplanar visualization of perimembranous, inlet, or muscular defects; color Doppler refines shunt direction and significance.	Limited role in isolated small VSD; mainly adjunctive in complex anatomy.
**ASD/primum ASD spectrum**	Early four-chamber abnormalities and abnormal interatrial flow may suggest major septal defects in expert hands.	Better structural definition of primum ASD and associated AV canal features; venous anomalies may become clearer.	Usually limited; may support assessment of associated extracardiac or complex cardiac findings.
**TOF/conotruncal defects**	Outflow-tract and 3VT abnormalities, overriding aorta, large VSD, abnormal vessel proportions, indirect markers such as increased NT or abnormal DV flow.	Confirmation of conotruncal anatomy, RV outflow obstruction severity, great artery relationships, and associated lesions.	Useful adjunct when ultrasound is suboptimal or anatomy is complex.
**CoA/arch anomalies**	Suspicion based on isthmus and arch hypoplasia, valve and ventricular disproportion, and abnormal ratios.	Detailed arch measurements, CSAi, I/D ratio, DA index, 3VT abnormalities, and branch pattern assessment.	Helpful when echocardiography is inconclusive or branch anatomy is difficult to define.
**Venous return anomalies**	Additional vessel in 3VV/3VT or indirect signs may suggest PLSVC; TAPVC/PAPVC detection remains challenging.	Coronary sinus dilation, abnormal venous confluence, absent pulmonary venous entry to the left atrium, vertical vein, or retroatrial findings.	Useful adjunct in equivocal cases or poor sonographic windows, especially for systemic venous anomalies.

## Data Availability

The data presented in this study are available upon request from the corresponding author.

## Abbreviations

The following abbreviations have been used in the main text:

AI	artificial intelligence
AHA	American Heart Association
ALSA	aberrant left subclavian artery
ARSA	aberrant right subclavian artery
ASD	atrial septal defect
AV	atrioventricular
AVSD	atrioventricular septal defect
BF-STIC	B-flow spatiotemporal image correlation
CHD	congenital heart disease
CNV	copy-number variation
CoA	coarctation of the aorta
CSAi	carotid–subclavian artery index
DA	distal aortic arch
DS	Down syndrome
DV	ductus venosus
ENG	endoglin gene
FLNA	filamin A gene
3D	three-dimensional
3VV	three-vessel view
3VT	three-vessel and trachea view
4D	four-dimensional
HMGN1	high-mobility group nucleosomebinding domain 1
I/D	isthmus-to-ductal ratio
iPSC	induced pluripotent stem cell
JACC	Journal of the American College of Cardiology
KCNH2	potassium voltage-gated channel subfamily H member 2 gene
LV	left ventricle/ventricular
ML	machine learning
MPES	mid-pharyngeal endothelial strand
MRI	magnetic resonance imaging
NIPT	non-invasive prenatal testing
NOTCH	neurogenic locus notch homolog signaling
NT	nuchal translucency
OFT	outflow tract
PAPVC	partial anomalous pulmonary venous connection
PDA	patent ductus arteriosus
PLSVC	persistent left superior vena cava
SVC	superior vena cava
STR	short tandem repeat
STIC	spatiotemporal image correlation
T21	trisomy 21
TAPVC	total anomalous pulmonary venous connection
TAO-DAO	transverse aortic arch–descending aorta angle
TOF	tetralogy of Fallot
TR	tricuspid regurgitation
TV/MV	tricuspid valve-to-mitral valve diameter ratio
US	ultrasound
VSD	ventricular septal defect
